# Decoding the Absolute Stoichiometric Composition and Structural Plasticity of α-Carboxysomes

**DOI:** 10.1128/mbio.03629-21

**Published:** 2022-03-28

**Authors:** Yaqi Sun, Victoria M. Harman, James R. Johnson, Philip J. Brownridge, Taiyu Chen, Gregory F. Dykes, Yongjun Lin, Robert J. Beynon, Lu-Ning Liu

**Affiliations:** a Institute of Systems, Molecular and Integrative Biology, University of Liverpool, Liverpool, United Kingdom; b Centre for Proteome Research, Institute of Integrative Biology, University of Liverpool, Liverpool, United Kingdom; c GeneMill, Institute of Integrative Biology, University of Liverpool, Liverpool, United Kingdom; d National Key Laboratory of Crop Genetic Improvement and National Center of Plant Gene Research, Huazhong Agricultural University, Wuhan, China; e College of Marine Life Sciences, and Frontiers Science Center for Deep Ocean Multispheres and Earth System, Ocean University of China, Qingdao, China; McMaster University

**Keywords:** CO_2_-concentrating mechanisms, absolute quantification, bacterial microcompartment, carbon fixation, carboxysome, mass spectrometry, protein organelle, protein stoichiometry

## Abstract

Carboxysomes are anabolic bacterial microcompartments that play an essential role in carbon fixation in cyanobacteria and some chemoautotrophs. This self-assembling organelle encapsulates the key CO_2_-fixing enzymes, Rubisco, and carbonic anhydrase using a polyhedral protein shell that is constructed by hundreds of shell protein paralogs. The α-carboxysome from the chemoautotroph Halothiobacillus neapolitanus serves as a model system in fundamental studies and synthetic engineering of carboxysomes. In this study, we adopted a QconCAT-based quantitative mass spectrometry approach to determine the stoichiometric composition of native α-carboxysomes from H. neapolitanus. We further performed an in-depth comparison of the protein stoichiometry of native α-carboxysomes and their recombinant counterparts heterologously generated in Escherichia coli to evaluate the structural variability and remodeling of α-carboxysomes. Our results provide insight into the molecular principles that mediate carboxysome assembly, which may aid in rational design and reprogramming of carboxysomes in new contexts for biotechnological applications.

## INTRODUCTION

Bacterial microcompartments (BMCs) are self-assembling proteinaceous organelles that are widespread among bacterial phyla (1, 2). The BMC is composed of a virus-like polyhedral protein shell that sequesters a series of enzymes to segregate their metabolic processes from the cytoplasm and provide specific local microenvironments to favor enzymatic activities (3–6). Increasing evidence highlights the significant roles of BMCs in enhancing the metabolism of various carbon sources, alleviating metabolic cross talk, and encapsulating toxic/volatile metabolites (7–9).

Carboxysomes are anabolic BMCs for autotrophic CO_2_ fixation in all identified cyanobacteria and some chemoautotrophs (3, 6, 10–12). They encase the CO_2_-fixing enzymes, ribulose-1,5-bisphosphate carboxylase oxygenase (Rubisco) and carbonic anhydrase (CA), using a semipermeable shell, which allows the passage of negatively charged HCO_3_^−^ and ribulose 1,5-bisphosphate (RuBP) and probably preclude O_2_ influx and leakage of CO_2_ from the carboxysome to the cytoplasm (13–15). In the carboxysome lumen, HCO_3_^−^ is dehydrated to CO_2_ by CA, ensuring elevated CO_2_ levels around Rubisco to facilitate Rubisco carboxylation and reduce wasteful photorespiration (16, 17). Collectively, the intriguing self-assembly and selective permeability features of carboxysomes provide the structural basis for enhanced CO_2_ assimilation and substantial contributions to global primary production (11, 18, 19).

**FIG 1:**
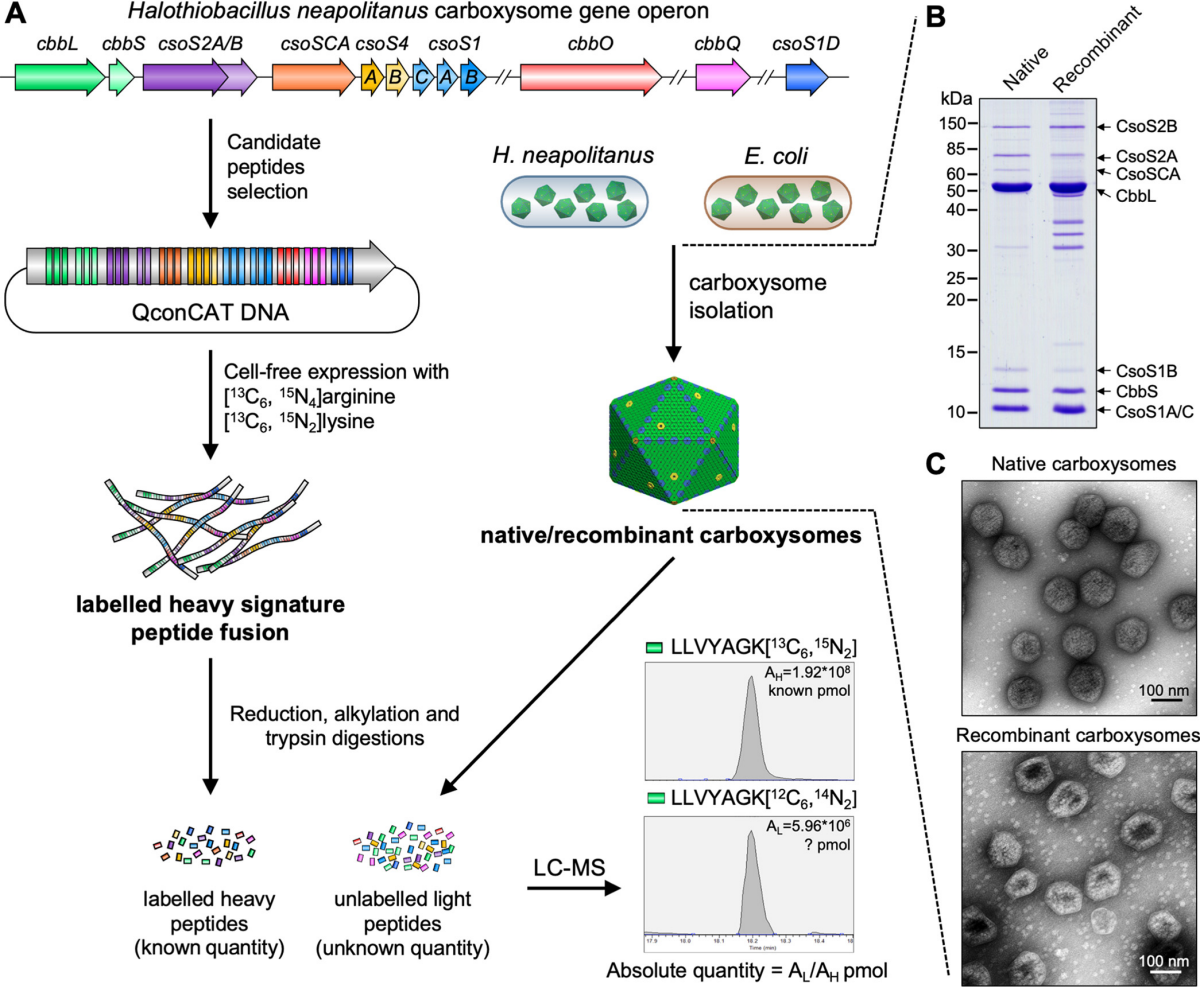
Schematic overview of QconCAT strategy. (A) A QconCAT was designed to encode concatenations of tryptic peptides from the *H. neapolitanus* α-carboxysome proteins, together with intervening peptide sequences that recapitulate the primary sequence context of the analyte peptides in the native proteins The QconCAT gene was expressed by cell-free synthesis and labeled with [^13^C_6_,^15^N_4_]arginine and [^13^C_6_,^15^N_2_]lysine. The purified and quantified QconCAT was added to four replicate samples of isolated native/recombinant α-carboxysomes from *H. neapolitanus*/E. coli. The absolute abundance and stoichiometry of the carboxysomal proteins were calculated by comparison of the area of the standard and analyte precursor ion chromatograms. A peptide for CbbQ, LLVKAGK, is shown here as an example. (B) SDS-PAGE of isolated native/recombinant α-carboxysomes showing the majority bands of α-carboxysome proteins. The protein bands with sizes between 15 and 40 kDa observed in recombinant carboxysomes might be cytoskeletal and membrane(-associated) proteins from E. coli as indicated by mass spectrometry (Data Set S1). (C) EM images of isolated native/recombinant α-carboxysomes.

According to the forms of encapsulated Rubisco and protein composition, carboxysomes can be categorized into two subclasses, α- and β-carboxysomes (11, 20). The α-carboxysome of the chemoautotrophic bacterium Halothiobacillus neapolitanus has been chosen as a model carboxysome in fundamental studies and synthetic engineering. The genes encoding α-carboxysome-related proteins are clustered mainly in the *cso* operon in the H. neapolitanus genome (Fig. 1). The shell is constructed by six types of paralogous proteins, including the hexameric proteins (BMC-H) CsoS1A, CsoS1B, and CsoS1C, which tile the major facet of shells, the pentamers (BMC-P) CsoS4A and CsoS4B, which sit at the vertices, and the trimeric pseudohexamer (BMC-T) CsoS1D, which possesses a larger central pore than other shell proteins and which was proposed to play a role in mediating the passage of large metabolite molecules, such as RuBP and 3-phosphoglycerate (3-PGA) (14, 21–23). Among the BMC-H proteins, CsoS1A and CsoS1C have a high sequence similarity, differing in only 2 amino acids out of 98 (24, 25), whereas CsoS1B contains a 12-residue C-terminal extension (24). The cargo enzymes include Rubisco and CA. Rubisco is assembled by the large and small subunits CbbL and CbbS, which form an L_8_S_8_ hexadecamer. CsoSCA acts as the functional CA in the α-carboxysome, existing as a dimer (26), and was suggested to associate with the shell inner surface (15, 27). The linker protein CsoS2 in the *H. neapolitanus* α-carboxysome has two isoforms: a shorter polypeptide, CsoS2A (C-terminally truncated), and a full-length CsoS2B, translated via programmed ribosomal frameshifting (28). CsoS2A and CsoS2B share the middle region and the N-terminal domain, which binds with Rubisco and induces Rubisco condensation (29). The C terminus of CsoS2B, which is absent in CsoS2A, is presumed to bind with the shell and can serve as an encapsulation peptide to recruit nonnative cargos (27, 30). In addition, CbbO and CbbQ function as the Rubisco activases, forming a bipartite complex comprising one CbbQ hexamer and one CbbO monomer, to remove inhibitors from the Rubisco catalytic site to restore its carboxylation (31–35).

10.1128/mbio.03629-21.1 DATA SET S1 Mass spectrometry protein analysis of purified native α-carboxysomes from *H. neapolitanus* and recombinant *H. neapolitanus* α-carboxysomes from E. coli. Download Data Set S1, XLSX file, 0.08 MB. Copyright © 2022 Sun et al. 2022 Sun et al. https://creativecommons.org/licenses/by/4.0/ This content is distributed under the terms of the Creative Commons Attribution 4.0 International license.

Given the significance of metabolic improvement and synthetic engineering potential, substantial efforts have been made to uncover the assembly and structural principles of carboxysomes. However, our knowledge about the accurate stoichiometric composition of carboxysomes, which plays an essential role in determining their size, shape, structural integrity, permeability, and catalytic performance (36), is still primitive. Label-free quantitative mass spectrometry has been used to determine the relative content of protein compositions within the BMCs (37–40). Furthermore, our recent work has applied mass spectrometry-based absolute quantification and a QconCAT (quantification concatemer of standard peptides) strategy to examine the precise stoichiometric composition of 1,2-propanediol utilization (PDU) metabolosomes from Salmonella enterica serovar Typhimurium LT2 (41). In addition, fluorescence labeling and microscopic imaging have been utilized to characterize the protein stoichiometry of β-carboxysomes from the cyanobacterium Synechococcus elongatus PCC 7942 (Syn7942) (42). However, the precise stoichiometric composition of α-carboxysomes has not been well characterized, despite the crude estimates based on protein electrophoresis profiles reported in previous studies (22, 43, 44).

In this study, we performed absolute quantification of protein components within native α-carboxysomes from *H. neapolitanus* and recombinant α-carboxysomes produced in Escherichia coli, using QconCAT-assisted quantitative mass spectrometry (MS) in combination with biochemical analysis, electron microscopy (EM), and enzymatic assays. Our results shed light on the molecular principles underlying the assembly and structural plasticity of α-carboxysomes and provide essential information required for design and engineering of carboxysomes in synthetic biology.

## RESULTS

### Quantifying the protein stoichiometry of native α-carboxysomes from *H. neapolitanus*.

The QconCAT-assisted mass spectrometry approach permitted a precise quantification of the absolute abundance of proteins (45–47). This approach has been recently applied to quantify the stoichiometric composition of protein components within the PDU metabolosome (41). To determine the stoichiometry of α-carboxysome components, native α-carboxysomes were first isolated from *H. neapolitanus* using sucrose gradient ultracentrifugation (see Fig. S1A in the supplemental material). Sodium dodecyl sulfate-polyacrylamide gel electrophoresis (SDS-PAGE) indicated that CsoS2A/B, CbbL/S, and CsoS1A/B/C are the major α-carboxysomes (Fig. S1B). NADH-coupled CO_2_ fixation activity assays confirmed the functionality of isolated α-carboxysomes, with a measured carbon fixation *V_max_* of 2.96 ± 0.09 μmol·mg^−1^·min^−1^ and *K_m_* for RuBP [*K_m_*_(RuBP)_] at 0.20 ± 0.02 mM (*n *= 4) (Fig. S1C). EM showed that the isolated α-carboxysomes form an intact and canonical polyhedral shape, with an average diameter of 124.6 ± 9.6 nm (*n *= 272) (Fig. S1D and E), consistent with previous results (31, 48, 49).

10.1128/mbio.03629-21.2 FIG S1 Purification and characterization of native and recombinant α-carboxysomes from *H. neapolitanus* and E. coli, respectively. (A) Sucrose gradient for carboxysome fractions. The milky band between 35% and 50% sucrose fraction interface consists of enriched carboxysomes. (B) SDS-PAGE of purified carboxysomes isolated from four replicate batches of culture showing the majority bands of α-carboxysome proteins. See also Fig. S3. (C) Carbon fixation kinetics as a function of RuBP concentrations (0 mM, 0.06 mM, 0.13 mM, 0.25 mM, 0.5 mM, 1 mM, and 2 mM) revealed that native and recombinant carboxysomes possess *V_max_* at 2.96 ± 0.09 and 2.07 ± 0.12 μmol·mg^−1^·min^−1^ and *K_m_*_(RuBP)_ at 0.20 ± 0.02 and 0.08 ± 0.02 mM, respectively. Data are presented as means ± SD from four independent biological replicates. (D) TEM images of purified native and recombinant carboxysomes. (E) Boxplot distribution for diameters of purified native and recombinant carboxysomes, at 124.6 ± 9.6 nm (*n *= 272) and 131.8 ± 18.0 nm (*n *= 152), respectively. Significant difference of average diameter was confirmed with Student *t* test (*P < *0.05). (F) Analysis of the shell thickness of native and recombinant α-carboxysomes. The shell thicknesses of native and recombinant α-carboxysomes are 5.3 ± 0.6 nm and 5.5 ± 0.8 nm, respectively (*n *= 100), implicating the single-layer shell architecture. The profile region for measurements is marked by red lines (scale bar = 50 nm). (G) Recombinant α-carboxysome expression cassette containing *cso* operon and *cbbL/S*, plus BMC-T protein-encoding gene *csoS1D* on the pBAD33 vector. Download FIG S1, JPG file, 1.7 MB. Copyright © 2022 Sun et al. 2022 Sun et al. https://creativecommons.org/licenses/by/4.0/ This content is distributed under the terms of the Creative Commons Attribution 4.0 International license.

To establish the accurate stoichiometry of all proteins within the isolated α-carboxysomes, we created a QconCAT (47, 50) to yield a series of stable isotope-labeled peptides as internal standards. The carboxysome preparations were mixed with a known amount of QconCAT and codigested, yielding analyte and standard peptides, differing only in the number of heavy atom centers. This codigest of analyte and standard was separated by nanoflow high-resolution liquid chromatography, coupled to high-resolution mass spectrometry (LC-MS) (Fig. 1 and Fig. S2). The QconCAT encoded three unique peptides for the CbbL, CbbS, CsoSCA, CbbO, CbbQ, CsoS1D, CsoS4A, and CsoS2AB shared region, two peptides for the CsoS2B and CsoS1ABC shared region, and a single peptide for the CsoS1B and CsoS1AC shared region (Fig. S2A and Table S1). Due to the high sequence similarity, CsoS1A and CsoS1C could not be distinguished in this QconCAT design. The QconCAT also encoded peptides to quantify the form II Rubisco CbbM. Since CbbM was presumed not to be a component of the *H. neapolitanus* α-carboxysome (51), we used CbbM as a reference to validate the quality of α-carboxysome isolation. The QconCAT gene encoding these peptide candidates was assembled from Qbricks using the ALACATs assembly strategy (47), to yield the QconCAT DNA sequence (Table S2). The QconCAT was then produced by cell-free synthesis (52) in the presence of stable isotope-labeled lysine and arginine, purified, and validated by SDS-PAGE and mass spectrometry (Fig. S2B).

10.1128/mbio.03629-21.3 FIG S2 Structure and expression of the *H. neapolitanus* carboxysome QconCAT. (A) Schematic representation of the quantification concatamer, for quantification of proteins of interest. Thirty-five peptides in QconCAT are represented by blue boxes. Values for the [M + 2H^2+^] *m/z* peptide ions for the unlabeled QconCAT are aligned above each peptide (green text). The nontarget quantification peptides (GluFib and cMyc) and the hexahistidine tag for QconCAT purification are shaded in red and orange, respectively. (B) SDS-PAGE analysis of QconCAT expression and purification. The coding sequence of QconCAT peptide was subcloned into the cell-free expression vector pEU-E01-MCS (left). QconCAT was prepared by wheat germ cell-free synthesis in the presence of [^13^C_6_,^15^N_4_]arginine and [^13^C_6_,^15^N_2_]lysine and purified by virtue of the hexahistidine tag (right). The QconCAT is indicated by the arrow. Download FIG S2, JPG file, 1.2 MB. Copyright © 2022 Sun et al. 2022 Sun et al. https://creativecommons.org/licenses/by/4.0/ This content is distributed under the terms of the Creative Commons Attribution 4.0 International license.

10.1128/mbio.03629-21.6 TABLE S1 Peptides derived from tryptic proteolysis of the QconCAT for carboxysome protein quantification. The flanking sequences that recapitulate the true native primary sequence context, together with additional sequences that are derived from the loop assembly synthesis of the QconCAT, are written in gray. Download Table S1, DOCX file, 0.05 MB. Copyright © 2022 Sun et al. 2022 Sun et al. https://creativecommons.org/licenses/by/4.0/ This content is distributed under the terms of the Creative Commons Attribution 4.0 International license.

10.1128/mbio.03629-21.7 TABLE S2 DNA and protein sequences of the carboxysome QconCAT peptide. Download Table S2, DOCX file, 0.04 MB. Copyright © 2022 Sun et al. 2022 Sun et al. https://creativecommons.org/licenses/by/4.0/ This content is distributed under the terms of the Creative Commons Attribution 4.0 International license.

From the LC-MS/MS traces, the peptide precursor ions for analyte and standards were isolated and their relative areas were quantified using Skyline (53). This was repeated with four independent preparations of carboxysomes (Fig. 1A and Fig. S3). All carboxysomal proteins were detected in the isolated carboxysomes, whereas CbbM was not detectable in the isolated samples; the carboxysomal proteins accounted for 99.5% ± 0.2% of the total proteins in the samples (Fig. S4A). These results confirm the high purity and the structural and functional integrity of isolated carboxysomes.

10.1128/mbio.03629-21.4 FIG S3 SDS-PAGE of purified carboxysomes from *H. neapolitanus* and E. coli with four biological replicates prepared for quantification by QconCAT mass spectrometry. The 10 μg of proteins was loaded per well. The protein bands with sizes between 15 and 40 kDa observed in recombinant carboxysomes might be cytoskeletal and membrane(-associated) proteins from E. coli as suggested by mass spectrometry (Data Set S1). Similar protein bands have been visualized in the isolated synthetic α-carboxysomes and empty carboxysome shells as reported previously (T. Chen, Y. Fang, Q. Jiang, G. F. Dykes, et al., ACS Synth Biol 11:154–161, 2022, https://doi.org/10.1021/acssynbio.1c00311; T. Li, Q. Jiang, J. Huang, C. M. Aitchison, et al., Nat Commun 11:5448, 2020, https://doi.org/10.1038/s41467-020-19280-0). Download FIG S3, JPG file, 0.6 MB. Copyright © 2022 Sun et al. 2022 Sun et al. https://creativecommons.org/licenses/by/4.0/ This content is distributed under the terms of the Creative Commons Attribution 4.0 International license.

10.1128/mbio.03629-21.5 FIG S4 Evaluation of QconCAT and label-free quantification. (A) Protein profile of all identified proteins in label-free quantification. In native carboxysome samples, McdAB-like proteins are both detected. The full protein list is provided in Data Set S1. (B) Comparison of QconCAT and label-free quantification. Quantification was normalized to equal total protein quantity. Proteins with an abundance difference greater than 30% from average are labeled in red. (C) Quantification of all QconCAT candidate peptides for *H. neapolitanus* carboxysomes. Overall good agreements were found within candidate peptides for the same protein, with the exception of peptide 3 of CsoSCA (34% ± 4% lower than the other two) and CbbO (21% ± 3%) in native carboxysome samples. Different colors represent distinct QconCAT peptides that were used to quantify the same carboxysome protein, which is listed in Table S1. Data are shown as means ± SD from four independent biological replicates. Download FIG S4, JPG file, 1.4 MB. Copyright © 2022 Sun et al. 2022 Sun et al. https://creativecommons.org/licenses/by/4.0/ This content is distributed under the terms of the Creative Commons Attribution 4.0 International license.

We quantified the abundance of protein components within one *H. neapolitanus* carboxysome structure, based on the shell surface area of a typical icosahedron (54) and the average carboxysome size (124.6 ± 9.6 nm; *n *= 272) measured in EM (Fig. 1C, Table 1, and Table S3; see details in Materials and Methods). The results revealed that the most abundant proteins in the *H. neapolitanus* α-carboxysome are CsoS1AC hexamers (863 copies), followed by Rubisco (447 copies, estimated by the CbbL content, as CbbL subunits contain all the catalytic sites per Rubisco), CsoS2A (248 copies), CsoS2B (192 copies), CsoS1B hexamers (112 copies), and 58 copies of CsoSCA dimers. The *H. neapolitanus* α-carboxysome has a molecular weight (MW) of ∼346 MDa and the Rubisco enzymes account for ∼66% of the total MW. The hexameric shell proteins CsoS1A/C and CsoS1B make up ∼17.1% of the total MW. Additionally, about 11 copies of CsoS4A/B pentamers (CsoS4A, 9; CsoS4B, 2) are integrated within the α-carboxysome. CsoS1D pseudohexamers have a low abundance in the shell, with ∼3 copies per carboxysome. Moreover, the linker proteins, CsoS2A and CsoS2B, account for 13.5% of the total MW.

**TABLE 1:** QconCAT quantification of protein components in native α-carboxysomes from *H. neapolitanus* and recombinant α-carboxysomes from E. coli

Category	Protein	Structure of functional unit (reference[s])	MW	% total protein by wt,^*a*^ native/recombinant	Units of monomeric protein per carboxysome, native/recombinant	Functional units of multimer per carboxysome, native/recombinant
Structural proteins	CsoS1AC	Hexamer (24, 74)	10.0 kDa	14.9 ± 1.1/17.8 ± 0.8	5,175 ± 378/6,003 ± 268	863 ± 63/1,001 ± 45
	CsoS1B	Hexamer^*b*^	11.3 kDa	2.2 ± 0.5/1.6 ± 0.2	673 ± 148/473 ± 57	112 ± 25/79 ± 9
	CsoS1D	Pseudohexamer (23)	23.5 kDa	0.12 ± 0.0/0.03 ± 0.0	17 ± 1/5 ± 0	3 ± 0/1 ± 0
	CsoS4A	Pentamer (75)	8.9 kDa	0.1 ± 0.0/0.1 ± 0.0	44 ± 2/32 ± 4	9 ± 0/6 ± 1
	CsoS4B	Pentamer (76)	8.8 kDa	0.03 ± 0.0/0.01 ± 0.0	11 ± 2/4 ± 3	2 ± 0/1 ± 1
	CsoS2A	Monomer (27, 29)	92.4 kDa	6.6 ± 1.3/8.4 ± 0.3	248 ± 47/305 ± 9	248 ± 47/305 ± 9
	CsoS2B	Monomer (27, 29)	124.2 kDa	6.9 ± 0.5/9.2 ± 0.5	192 ± 15/249 ± 13	192 ± 15/249 ± 13
Catalytic proteins	CbbL	L_8_S_8_ hexadecamer (29)	52.6 kDa	54.4 ± 2.3/53.4 ± 4.0	3,576 ± 151/3,408 ± 252	447 ± 19/426 ± 32
	CbbS	L_8_S_8_ hexadecamer (29)	12.9 kDa	11.7 ± 0.6/9.4 ± 0.4	3,161 ± 151/2,449 ± 96	395 ± 19/306 ± 12
	CsoSCA	Dimer (26)	57.3 kDa	1.9 ± 0.1/0.1 ± 0.0	117 ± 7/4 ± 1	58 ± 4/2 ± 1
	CbbQ	Hexamer (31)	30.1 kDa	0.76 ± 0.05/—^*c*^	88 ± 5/—	15 ± 1/—
	CbbO	Monomer (31, 33)	88.6 kDa	0.39 ± 0.03/—	15 ± 1/—	15 ± 1/—
Intact native α-carboxysome			346 MDa			
Intact recombinant α-carboxysome			336 MDa			

a Data are presented as means ± SD from four biological replicates. The quantity of each protein in each biological replicate was determined as the average quantity of correlated QconCAT peptides (see Materials and Methods and Table S1).

b Based on the structural similarity with CsoS1A/C.

c —, No corresponding protein identified in recombinant carboxysomes.

10.1128/mbio.03629-21.8 TABLE S3 Calculation of carboxysome surface area, shell hexamer content, and carboxysome diameter. *CsoS1 and CsoS4 width/area obtained from previous publications (S. Tanaka, C. A. Kerfeld, M. R. Sawaya, F. Cai, et al., Science 319:1083–1086, 2008, https://doi.org/10.1126/science.1151458; Y. Tsai, M. R. Sawaya, G. C. Cannon, F. Cai, et al., PLoS Biol 5:e144, 2007, https://doi.org/10.1371/journal.pbio.0050144). &Assumed packing densities of 74% (Kepler packing) (L. Whitehead, B. M. Long, G. D. Price, M. R. Badger, Plant Physiol 165:398–411, 2014, https://doi.org/10.1104/pp.114.237941) in the proposed carboxysome model based on the measured carboxysome diameters. Download Table S3, DOCX file, 0.04 MB. Copyright © 2022 Sun et al. 2022 Sun et al. https://creativecommons.org/licenses/by/4.0/ This content is distributed under the terms of the Creative Commons Attribution 4.0 International license.

Approximately 15 copies of CbbQO complexes, each composed of one CbbQ hexamer and one CbbO monomer, were identified in the carboxysome, indicating that the CbbQO complex is a structural component of native α-carboxysomes in *H. neapolitanus*. Consistently, CbbQ has been indicated to be tightly associated with the *H. neapolitanus* carboxysome shell (31), and CbbQO can be incorporated into recombinant α-carboxysomes (35). Likewise, our mass spectrometry results showed the presence of McdAB-like proteins in purified native α-carboxysomes (Fig. S4A and Data Set S1), implicating the association of McdAB-like proteins with α-carboxysomes, which was proposed to ensure proper distribution of α-carboxysomes in *H. neapolitanus* and carboxysome inheritance during cell division (55). Some chemoautotrophs, including *H. neapolitanus*, contain form II Rubisco (CbbM) and its activases CbbQ2 and CbbO2 (32). These proteins were not detected in the purified carboxysomes (Data Set S1), suggesting that they are not the organizational components of or associated with the α-carboxysomes in *H. neapolitanus*.

### Stoichiometric composition of recombinant α-carboxysomes.

Previous studies have demonstrated that heterologous engineering of the *H. neapolitanus* α-carboxysomes could result in functional α-carboxysome structures (21, 35, 56, 57). To verify the compositional similarity between native and recombinant α-carboxysomes, we reconstituted *H. neapolitanus* α-carboxysomes by expressing the *cso* operon with *csoS1D* using an arabinose-inducible pBAD33 vector in E. coli (Fig. S1A and G). SDS-PAGE revealed overall similar contents of protein components within the isolated native and recombinant α-carboxysomes, except for a reduction in the CsoSCA content in recombinant carboxysomes (Fig. S1B and S3). Carbon fixation kinetics as a function of RuBP concentrations confirmed the function of recombinant α-carboxysomes, with a *V_max_* of 2.07 ± 0.12 μmol·mg^−1^·min^−1^ (*n *= 4) and a *K_m_*_(RuBP)_ of 0.08 ± 0.02 mM (*n *= 4), though both were lower than those of native α-carboxysomes (Fig. S1C). EM indicated that recombinant α-carboxysomes possess a polyhedral shape and an average diameter of 131.8 ± 18.0 nm (*n *= 152), slightly larger than native α-carboxysomes (Fig. S1D and E). Analysis of EM images showed that both native and recombinant α-carboxysomes possess single-layer shells (5.3 ± 0.6 nm and 5.5 ± 0.8 nm, respectively; *n *= 100 [Fig. S1F]), consistent with previous observations (37).

**FIG 2:**
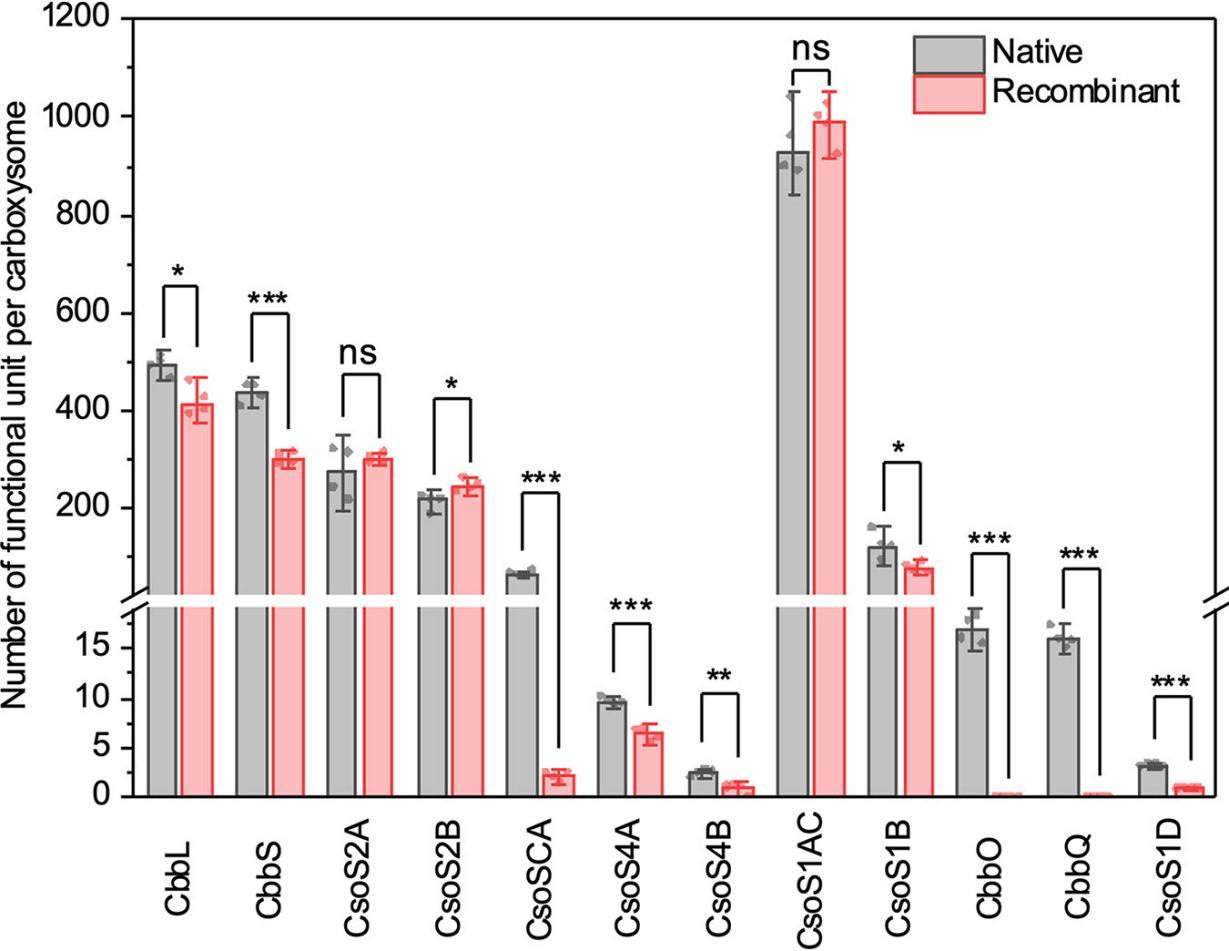
Stoichiometry comparison of native and recombinant carboxysomes. Absolute quantification using QconCAT standardization was used to obtain subunit stoichiometry. Differences between native and recombinant α-carboxysomes are highlighted. See also Table 1. *, *P < *0.05; **, *P < *0.01; ***, *P < *0.001 using two-sample *t* test, equal variance not assumed (Welch correction). Data are presented as means ± SD from four independent biological replicates. ns, not significant.

Individual proteins in isolated recombinant α-carboxysomes were then quantified by mass spectrometry to retrieve the stoichiometric content of the two types of α-carboxysomes (Fig. 1, Table 1, and Table S3). Within the recombinant α-carboxysome, the most abundant proteins are CsoS1AC hexamers (1,001 copies), followed by Rubisco (426 copies), CsoS2A (305 copies), CsoS2B (249 copies), and CsoS1B hexamers (79 copies). The recombinant α-carboxysome has a molecular mass of ∼336 MDa and has reduced Rubisco copy numbers compared with the native α-carboxysome (*P < *0.05 [Fig. 2]). The content of CsoSCA in the recombinant α-carboxysome is reduced by 29-fold compared with that in the native α-carboxysome, resulting in only ∼2 CsoSCA dimers per recombinant α-carboxysome, consistent with SDS-PAGE analysis (Fig. S1B). The hexameric shell proteins, CsoS1AC and CsoS1B, account for 19.4% of the total MW in recombinant α-carboxysomes (Table 1). The CsoS1B content is reduced by ∼30% (79 copies) compared with that in native α-carboxysomes (112 copies; *P < *0.05; [Fig. 2]). There are, on average, 7 copies of pentameric proteins (CsoS4A, 6; CsoS4B, 1) in recombinant α-carboxysomes, less than the hypothetical 12 pentamers for a typical icosahedral structure. This suggests that some vertices are not capped by CsoS4 pentamers. Similar features have also been observed in β-carboxysomes and synthetic BMC shells (42, 58, 59), presumably providing a mechanism for regulating shell architecture and permeability. CsoS1D has ∼1 copy per recombinant α-carboxysome, less than that in the native α-carboxysome (*P < *0.001 [Table 1]). CsoS2A and CsoS2B have 305 and 249 copies, respectively, per recombinant α-carboxysome, collectively accounting for 17.6% of the total MW. CsoS2B has an increased content in the recombinant α-carboxysome compared to the native form (Fig. 2).

## DISCUSSION

In this study, we performed absolute quantification using QconCAT-based mass spectrometry to determine the stoichiometric composition of the *H. neapolitanus* α-carboxysomes, which represent a step toward gaining a comprehensive understanding of the structure and function of the model carboxysome.

The building components in the carboxysome have a wide range of abundances, from a few to thousands of copies per carboxysome. The major proteins could be visualized in protein gels, whereas some minor proteins were hardly visible (Fig. 1 and Fig. S2). In addition, shell protein paralogs often have similar molecular masses and therefore were not readily distinguishable in SDS-PAGE gels. These intrinsic characteristics made it difficult to obtain the accurate and complete stoichiometry of carboxysomes solely based on protein band profiling of protein electrophoresis and label-free MS quantification. The QconCAT approach is a very effective method for determination of subunit stoichiometry. Because all of the labeled standard peptides are released in equal amounts (completeness of digestion was confirmed), they form a valuable baseline against which the intensities of the analyte cognate peptides can be measured. In this study, we were able to quantify subunits over 3 to 4 orders of magnitude, with high accuracy. Because the approach relies on internal standards, it does not depend on specific properties of members of the protein complex, such as SDS-PAGE band intensity, or the intrinsic properties of individual peptides in label-free proteomics, although the correlation between QconCAT absolute quantification and label-free intensities in this instance was acceptable (Fig. S4).

Comparison of QconCAT and label-free quantification results illustrated notable deviations in the abundances of some carboxysomal proteins (Fig. S4B). The results demonstrate that label-free quantification could potentially lead to inaccurate estimates in the contents of CsoS1B, CsoSCA, CsoS4A, and CsoS4B and highlight the necessity of QconCAT-based quantification in studying the protein stoichiometric composition of BMCs. Moreover, the reliability of QconCAT quantification was evident by a great agreement between individual QconCAT peptides for the same carboxysome protein (Fig. S4C).

### Stoichiometric variability and structural plasticity of α-carboxysomes.

**FIG 3:**
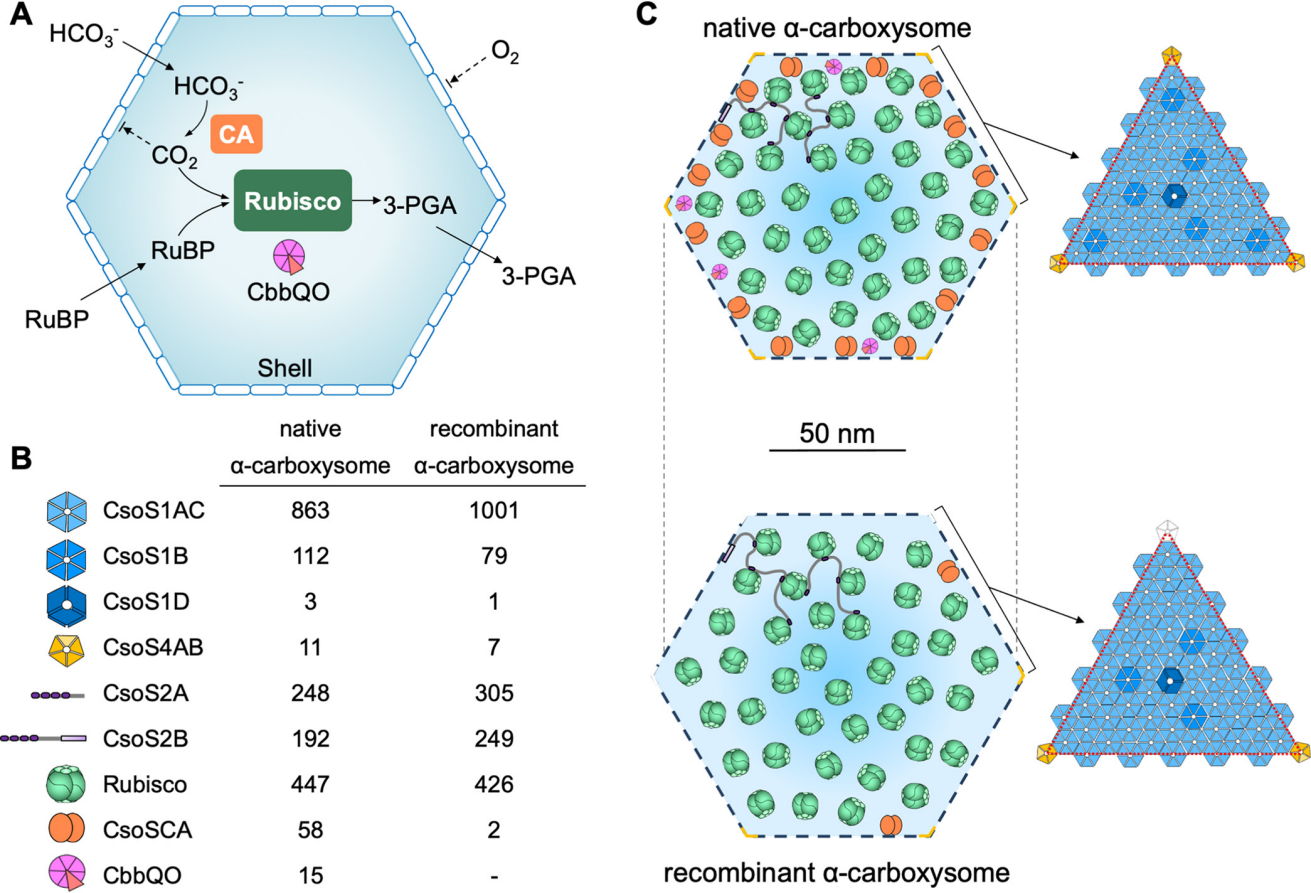
Structural models of *H. neapolitanus* α-carboxysomes. (A) Schematic of the pathways of carbon fixation in the α-carboxysome, including Rubisco activases CbbQO as the structural components; (B) stoichiometry of each structural component within native and recombinant α-carboxysomes (see Table 1); (C) schematic of native and recombinant α-carboxysome structures and shell organization. The numbers of proteins do not represent actual abundances and are only for illustration.

Characterization of the absolute stoichiometric compositions for native and recombinant carboxysomes provides insight into the organizational principles and plasticity of the *H. neapolitanus* α-carboxysome (Fig. 3). It becomes apparent that the BMC shells are amendable to integrate different copies or types of shell proteins, and the absence of specific components or the changes in the ratios of protein paralogs may not necessarily impede the overall shell assembly (41, 60–62). The total copy numbers of shell pentamers (CsoS4A and CsoS4B) are 11.0 for native α-carboxysomes and 7.1 for recombinant α-carboxysomes, both less than the 12 pentamers that are postulated to occupy all the vertices of a regular icosahedron (3, 7). These results elucidated that it is not a prerequisite to cap all the vertices with pentamers in a functional carboxysome. In support of this, polyhedral carboxysomes and BMC shells deficient in pentamers could still be formed (59, 60, 63, 64). Our previous study has also demonstrated that variable copies of CcmL pentamers are integrated into Syn7942 β-carboxysomes under different growth conditions (42). The lack of pentamers at some vertices might result in observable structural heterogeneity and reduced integrity of the entire α-carboxysomes (Fig. S1D).

Rubisco in carboxysomes was proposed to adopt a Kepler packing, filling maximally 74% of the internal carboxysome volume (54, 65). Quantification based upon the CbbL content indicates that the native *H. neapolitanus* α-carboxysome can accommodate approximately 447 copies of Rubisco (the CbbL/CbbS ratio is 8:7.3), in agreement with the theoretical estimation based on the Kepler packing (411 copies of Rubisco, Table S3). In contrast, recombinant α-carboxysomes encapsulate 426 Rubisco (the CbbL/CbbS ratio is 8:5.7), lower than the estimated copy number of 491 based on measured recombinant carboxysome size (Table S3). The various CbbL/CbbS ratios of Rubisco might affect accurate determination of Rubisco content and carboxylation activity within the α-carboxysome and merits further investigation. The recombinant α-carboxysomes have a greater diameter (131.8 nm) and shell/interior ratio (1:1) than those of native α-carboxysomes (124.6 nm and 0.8:1) (Table 2 and Fig. S1D and E). Our results suggest a lower level of Rubisco packing within recombinant α-carboxysomes (Fig. 3), presumably explaining the “darker centers” of recombinant carboxysomes observed in EM (Fig. 1C and Fig. S1D). Moreover, the perturbed formation of Rubisco (L_8_S_8_) as indicated by the changes in the CbbL/CbbS ratio has also been determined in recombinant carboxysomes (Table 2). Our results also showed that the Rubisco/CA (CbbL/CsoSCA) ratios vary drastically between native and recombinant α-carboxysomes (Table 2). It has been postulated that too little or too much carboxysomal CA activity, which could cause limited CO_2_ supply or substantial leakage of CO_2_, may interfere with CO_2_ fixation of carboxysomes (11). What caused the decrease in the CsoSCA content within recombinant α-carboxysomes remains to be investigated. It is possible that *csoSCA* was not strongly expressed in the E. coli host with its native expression element, such as ribosome binding site (RBS) from *H. neapolitanus*, or expressed CsoSCA proteins were not efficiently integrated into α-carboxysomes in the nonnative intracellular environment. Modification of CsoSCA expression, such as adjusting the promoter and RBS and optimizing the expression conditions, should be considered in future studies. Other changes that occurred in recombinant carboxysomes involve the increased content of CsoS1 shell proteins, the reduced CsoS1D abundance, as well as the absence of CbbQO (*cbbQ* and *cbbO* genes were not included in the expression construct) (Fig. 3). All these structural alternations may collectively result in the higher size variation of recombinant α-carboxysomes (Fig. S1E) and the discrepancy in the carbon fixation performance between native and recombinant α-carboxysomes (Fig. S1C).

**TABLE 2:** Stoichiometric ratios of protein components in α-carboxysomes^*a*^

Carboxysome proteins	Ratio	
	Native	Recombinant
CbbL/CbbS	8:7.3	8:5.7
CbbL/CsoS2	8:1	8:1.3
CbbL/CsoSCA	30.7:1	846:1
CsoS2A/CsoS2B	1.3:1	1.2:1
CsoS1 hexamer/CsoS2B	5.1:1	4.3:1
CsoS1AC/CsoS1B	7.7:1	12.7:1
CsoS4A/CsoS4B	4.1:1	8.1:1
CbbQ/CbbO	5.7: 1	NA
Shell proteins/internal enzymes	0.8:1	1:1
Hexamer/trimer	336.4:1	1,403.5:1
Hexamer/pentamer	89.1:1	133.9:1

a Interior proteins include CbbL, CbbS, and CsoS2; shell proteins include CsoS1 and CsoS4. There were 11 and 7.1 pentamers per unit of carboxysome for native and recombinant carboxysomes, respectively. NA, not applicable.

CsoS2 in α-carboxysomes serves as the scaffolding protein that interlinks Rubisco and shells (11, 27–29). The CbbL/CsoS2 ratios in native and recombinant α-carboxysomes remain within a narrow range between 8:1 and 8:1.3 (Table 2), implicating the correlation between Rubisco and CsoS2, which is fundamental for Rubisco condensation and internal packing. Likewise, the CsoS2A/CsoS2B ratio remains relatively unaltered in native (ratio of 1.3:1) and recombinant (ratio of 1.2:1) α-carboxysomes.

### Organizational features of diverse carboxysomes.

Peptide composition of the α-carboxysomes from the α-cyanobacterium Prochlorococcus marinus MED4 has been estimated based on standard protein gel profiles (22). The *H. neapolitanus* α-carboxysomes (∼125 nm in diameter) are larger in diameter than the *Prochlorococcus* α-carboxysomes (∼90 nm in diameter). Consistently, the *H. neapolitanus* α-carboxysome has a 1.8-fold-increased content of CsoS1 hexameric shell proteins (975 versus 539 hexamers) and encapsulates double the content of CsoSCA proteins (58 versus 29 dimers) and nearly 3-fold more Rubisco enzymes (447 versus 152 copies). The experimentally determined Rubisco content fits well with the theoretical estimate (411 copies for the *H. neapolitanus* carboxysome and 143 copies for the *Prochlorococcus* carboxysome), which were based on the carboxysome size and Kepler packing (54, 65). In contrast, PDU microcompartments, with diameters ranging from 90 to 130 nm, have a drastically higher shell/interior ratio (4.6:1) (41) than the *H. neapolitanus* α-carboxysome (0.8:1 [Table 2]), implying that Kepler packing of cargo enzymes is unlikely applicable to metabolosomes. The CsoSCA/CsoS1 ratio remains relatively constant in both native α-carboxysomes, presumably implicating their specific association within the carboxysomes. In contrast, CA in the Syn7942 β-carboxysomes, which is encoded by the *ccaA* gene, which is distant from the *ccm* operon, was demonstrated to have varying abundances per carboxysome under different environmental conditions (42). It remains to be investigated if the CsoSCA content in α-carboxysomes is subject to environmental modulation.

A noteworthy feature of the *Prochlorococcus* α-carboxysome is that it contains only the full length of CsoS2 without the short isoform as the *H. neapolitanus* counterpart does, which might lead to formation of carboxysomes with reduced Rubisco loading capacity and overall size. However, the Rubisco/CsoS2 ratios in the α-carboxysomes from *H. neapolitanus* and *Prochlorococcus* remain relatively comparable (1:1 and 1:1.1, respectively), indicative of a general mechanism for Rubisco encapsulation of α-carboxysomes. In the Syn7942 β-carboxysome, the ratios between Rubisco and the scaffolding protein CcmM varied in a range of 1:0.8 to 1:1.3, depending upon environmental conditions (42). Unlike the similar CsoS2A/CsoS2B ratios in native and recombinant α-carboxysomes, the CcmM35/CcmM58 ratios in the Syn7942 β-carboxysomes have a wide range, 1:1 to 11:1, and have been proved to be vital for carboxysome assembly (65, 66).

Carboxysomes are highly modular structures with the capacity of incorporating foreign cargos, representing an ideal system in synthetic biology (30). Advanced knowledge about the precise protein stoichiometry of functional carboxysomes and the approach to determine the stoichiometry of natural and synthetic carboxysomes developed in this study open the door toward reprogramming and compositional refinement of carboxysomes for metabolic enhancement and diverse biotechnological applications in new contexts (36). The QconCAT-based protein quantification technique could also be broadly used in the studies of diverse BMC paralogs and engineering of a variety of protein organelles in their native origins and heterologous organisms.

## MATERIALS AND METHODS

### Bacterial strains, growth conditions, and carboxysome production.

*H. neapolitanus* (Parker, Kelly and Wood ATCC 23641 C2) used in this work was acquired from the American Type Culture Collection (ATCC) as freeze-dried powder (77, 78). Stock cells were maintained in liquid ATCC medium 290 (78) or on ATCC 290 1.5% agar plates. For scale-up cultivation and carboxysome purification, a 5-mL seeding culture was inoculated in 5 L of Vishniac and Santer growth medium (67), which contained the following per liter: 10.0 g of Na_2_S_2_O_3_·5H_2_O, 4.0 g of KH_2_PO_4_, 4.0 g of K_2_HPO_4_, 0.8 g of MgSO_4_·7H_2_O, 0.4 g of NH_4_Cl, and 10.0 mL of Vishniac and Santer trace element solution [50.0 g of EDTA, 22.0 g of ZnSO_4_·7H_2_O, 5.54 g of CaCl_2_, 5.06 g of MnCl_2_·4H_2_O, 4.99 g of FeSO_4_·7H_2_O, 1.10 g of (NH_4_)_6_Mo_7_O_24_·4H_2_O, 1.57 g of CuSO_4_·5H_2_O, and 1.61 g of CoCl_2_·6H_2_O per L, pH 6.0] (67). Cell growth was maintained in a 5-L fermentor (BioFlo 115; New Brunswick Scientific, USA) at 30°C. The pH of the growth medium was monitored by a pH probe and was maintained at 7.6 by constant supplementation with 3 M KOH. Air supply was set at 500 L·min^−1^ for initial growth and reduced to 200 L·min^−1^ 24 h prior to harvesting. Agitation was kept at 250 to 300 rpm. The optical density at 600 nm (OD_600_) of the culture was checked daily, and the cells were harvested before the culture entered the stationary phase. For expression of recombinant carboxysomes, the entire *cso* operon, as designed on *pHnCBS1D* reported previously (21), was fused on a pBAD33 arabinose-inducible expression vector (68) using the Gibson assembly strategy (69) with Gibson assembly master mix from New England BioLabs (NEB). Primer sets used for assembly are listed in Table S5. For recombinant carboxysome expression in E. coli, seeding cultures containing chloramphenicol at a final concentration of 50 μg mL^−1^ were inoculated at 37°C in LB broth until reaching an OD_600_ at 0.6 and then scaled up for induction with 1 mM arabinose at 20°C overnight.

10.1128/mbio.03629-21.10 TABLE S5 Primer sets used for pBAD33-CBS1D construction. Download Table S5, DOCX file, 0.04 MB. Copyright © 2022 Sun et al. 2022 Sun et al. https://creativecommons.org/licenses/by/4.0/ This content is distributed under the terms of the Creative Commons Attribution 4.0 International license.

### Carboxysome purification from *H. neapolitanus* and E. coli.

Purification of α-carboxysome from *H. neapolitanus* was modified from the protocol described previously (70). The 5-L culture harvested from the bioreactor that contained *H. neapolitanus* cells and elemental sulfur sediments was first pelleted down at 12,000 × *g* for 10 min. The pellet containing both cells and elemental sulfur sediments was resuspended in 60 mL of TEMB buffer (10 mM Tris-HCl, 10 mM MgCl_2_, 20 mM NaHCO_3_, 1 mM EDTA [pH 8.0]) and subsequentially centrifuged at 300 × *g* for 15 min to sediment elemental sulfur. The supernatant was transferred to a new centrifugation tube, and cells were obtained by another round of centrifugation at 12,000 × *g* for 10 min. The resulting cell pellet was resuspended in 15 mL of TEMB buffer and incubated with egg lysosome (at a final concentration of 0.5 mg mL^−1^) for 1 h at 30°C before cell breakage by prewashed glass beads (150- to 212-μm glass beads, acid washed; Sigma-Aldrich) for 10 min (30-s beating and 30-s interval on ice). The cell extracts were further treated with 33% (vol/vol) B-PERII (Thermo Fisher Scientific, UK) and 0.5% (vol/vol) IGEPAL CA630 (Sigma-Aldrich) and placed on a rotary mixer for 2 h. The unbroken cells and large membrane debris were removed by centrifugation at 9,000 × *g* for 10 min. Crude carboxysome enrichment was pelleted at 48,000 × *g* for 30 min. The pellet was resuspended, briefly centrifuged at 9,000 × *g*, and then loaded to a step sucrose gradient (10%, 20%, 30%, 35%, 50%, and 60%) and ultracentrifuged at 105,000 × *g* for 35 min. The 3 mL of enriched carboxysome was harvested at 35% to 50% sucrose gradient fractions. Sucrose was removed by an additional round of ultracentrifugation after dilution with 30 mL of TEMB buffer. The pure carboxysome pellet was resuspended in 800 μL of TEMB buffer. Unless indicated otherwise, all procedures were performed at 4°C. The carboxysome purification from E. coli was performed according to the previous protocols (21, 35, 71), with modifications. E. coli cells were lysed with B-PER II bacterial protein extraction reagent (Thermo Fisher Scientific, UK) and treated with 0.5% (vol/vol) IGEPAL CA-630 detergent for 2 h. The following purification steps were the same as for the isolation of native carboxysomes from *H. neapolitanus* as described above.

### SDS-PAGE analysis.

SDS-PAGE analysis was performed following standard procedures. The 10 μg of purified carboxysomal samples or 100 μg of whole-cell fractions was loaded per well on 15% polyacrylamide gels and stained with Coomassie brilliant blue G-250 (Thermo Fisher Scientific, UK).

### Electron microscopy and data analysis.

Electron microscopy was carried out as described previously (37). The purified carboxysomes (∼4 mg mL^−1^) were stained with 3% uranyl acetate on carbon grids and then inspected with an FEI 120 kV Tecnai G2 Spirit BioTWIN transmission electron microscope (TEM) equipped with a Gatan Rio 16 camera. The diameters of carboxysomes were measured with ImageJ as described previously (37) and were statistically analyzed using OriginPro 2020b (OriginLab, MA).

### Rubisco activity assays.

Carbon fixation assay was carried out to determine carbon fixation capacities of purified native and recombinant carboxysomes as described previously using a 3-phosphoglycerate-dependent NADH oxidation-coupled enzyme system (21). For both native and synthetic samples, four biological replicates that were isolated from different culture batches were assayed at 30°C, initiated with the final concentrations of 0 mM, 0.06 mM, 0.13 mM, 0.25 mM, 0.5 mM, 1 mM, and 2 mM RuBP. The concentration of HCO_3_^−^ was set to 24 mM for all assays in this work.

### Design, cell-free expression, and purification of QconCAT standard.

Absolute quantification of the carboxysomal proteins was carried out by mass spectrometry analysis with stable isotope tryptic peptides as standards. The standard peptides were added in the form of a QconCAT, an artificial protein that is a concatenation of tryptic peptides in the same primary sequence context as the cognate analyte peptides (41, 50). For each analyte protein, up to three standard peptides were encoded in the QconCAT (Table S1). All candidate peptides were searched by BLAST against the *H. neapolitanus* and E. coli proteomes to ensure their uniqueness. Due to the high level of sequence similarity of CsoS1A/B/C, CsoS2A/B and CsoS4A/B, peptides representing shared sequences and unique sequences were included (Fig. S2). The DNA fragment encoded the above-mentioned peptides, together with sacrificial termini; N-terminal GluFib and cMyc (peptides to quantify the standard) and C-terminal His_6_ tag were created by the ALACAT/Qbrick assembly strategy as reported previously (47). The final DNA sequence (Table S2) was assembled into a pEU-E01 vector for cell-free expression using wheat germ lysate (CellFree Sciences Co., Ltd., Japan). Synthesis was completed with [^13^C_6_,^15^N_4_]arginine and [^13^C_6_,^15^N_2_]lysine (CK Isotopes Ltd., UK) using the WEPR8240H full-expression kit following default protocols (2BScientific Ltd., UK). The QconCAT peptide was purified with Ni-Sepharose suspension (GE Healthcare Ltd., UK) in centrifuge filters (Corning Costar Spin-X 0.45-μm-pore-size cellulose acetate membrane; Merck, UK) following standard protocols. Finally, the QconCAT peptide was precipitated and resuspended in 30 μL of 25 mM ammonium bicarbonate with 0.1% (wt/vol) RapiGest SF surfactant (Waters, UK) and protease inhibitors (Roche cOmplete mini-EDTA-free protease inhibitor cocktail; Merck, UK).

### Proteomic analysis.

For QconCAT quantification, native and synthetic carboxysome preparations, four replicates of each, were diluted to a final protein concentration of 2.5 μg per 40 μL of 25 mM NH_4_HCO_3_. QconCAT (approximately 5 pmol) and [Glu1]-fibrinopeptide B (5 pmol) were added, and samples were denatured using 2.5 μL of 1% (wt/vol) RapiGest (Waters, Manchester, UK) in 25 mM NH_4_HCO_3_ followed by incubation at 80°C for 10 min. Samples were reduced by the addition of 2.5 μL of 12 mM dithiothreitol in 25 mM NH_4_HCO_3_ and incubation at 60°C for 10 min. Alkylation was carried out by the addition of 2.5 μL of 36 mM iodoacetamide in 25 mM NH_4_HCO_3_ and incubation at room temperature for 30 min in the dark. Trypsin at 2.5 μL (200 ng in 25 mM NH_4_HCO_3_; Enzyme:Protease, 1:10) was added to each sample in a final digest volume of 50 μL. Samples were incubated at 37°C overnight. To remove residual RapiGest, digests were acidified by the addition of 0.5 μL of trifluoroacetic acid (TFA) followed by incubation at 37°C for 45 min. Samples were centrifuged at 17,200 × *g* for 30 min and transferred to fresh low-bind tubes.

LC-MS analyses were conducted on a QExactive HF quadrupole-Orbitrap mass spectrometer coupled to a Dionex Ultimate 3000 rapid-separation liquid chromatography (RSLC) nano-liquid chromatograph (Thermo Fisher Scientific, UK). Sample digest (2 μL) was loaded onto a trapping column (Acclaim PepMap 100 C_18_, 75 μm by 2 cm, 3-μm packing material, 100 Å) using a loading buffer of 0.1% (vol/vol) TFA–2% (vol/vol) acetonitrile in water for 7 min at a flow rate of 12 μL min^−1^. The trapping column was then set in-line with an analytical column (EASY-Spray PepMap RSLC C_18_, 75 μm by 50 cm, 2-μm packing material, 100 Å) and the peptides were eluted using a linear gradient of 96.2% buffer A (0.1% [vol/vol] formic acid)/3.8% buffer B (0.1% [vol/vol] formic acid in water/acetonitrile [80:20] [vol/vol]) to 50% A/50% B over 30 min at a flow rate of 0.3 μL min^−1^, followed by washing at 1% A/99% B for 5 min and reequilibration of the column to starting conditions. The column was maintained at 40°C, and the effluent was introduced directly into the integrated nano-electrospray ionization source operating in positive ion mode. The mass spectrometer was operated in MS-only mode, with survey scans between *m/z* 350 to 2,000 acquired at a mass resolution of 240,000 Full Width Half Maximum (FWHM) at *m/z* 200. The maximum injection time was 50 ms, and the automatic gain control was set to 3e6. The raw data files were incorporated in Skyline (53), and quantification was performed by determining the summed-peak area of the first three isotopes of each peptide. The quantity of each protein in each biological replicate was determined as the average quantity of correlated QconCAT peptides as shown in Table S1.

Additionally, preparations of the four native and synthetic carboxysomes were analyzed by label-free quantification. Carboxysomes were digested as described above but without [Glu1]-fibrinopeptide B and analyzed by LC-MS/MS as described above but with a data-dependent acquisition method consisting of a 60,000-resolution full-scan MS scan with Automatic Gain Control (AGC) set to 3e6 ions with a maximum fill time of 100 ms. The 16 most abundant peaks per full scan were selected for Higher Energy Collisional Dissociation (HCD) MS/MS (30,000 resolution, AGC set to 1e5 ions with a maximum fill time of 45 ms) with an ion selection window of 2 *m/z* and normalized collision energy of 30%. Ion selection excluded singularly charged ions and ions with a charge state equal to or greater than +6. To avoid repeated selection of peptides for fragmentation, the program used a 60-s dynamic exclusion window. The raw data files were imported into Progenesis QI for Proteomics v4. The chromatograms are aligned and normalized prior to label-free quantification. Peptide identification was performed by Mascot (v2.7; Matrix Science, UK) against the UniProt *H. neapolitanus* database (UP000009102; 2,353 sequences) and E. coli database (UP000000625; 4,438 sequences). A precursor mass tolerance of 10 ppm and a fragment ion mass tolerance of 0.01 Da were applied with dynamic modifications of ^13^C_6_^15^N_2_ K, ^13^C_6_^15^N_4_ R, and oxidation (M) and with the static modification of carbamidomethylation (C).

For single-carboxysome quantitative normalization, relative quantifications from QconCAT were normalized based on the 12-pentamer coverage or hexameric and pentameric protein coverage within a single-layer shell (Table S4). Twelve-pentamer normalization was done via assuming 60 copies of monomeric CsoS4A and CsoS4B in sum per carboxysome. For shell coverage normalization, the shell surface area is first calculated using TEM measured diameter with the following formula:

$$A_{f}=5\sqrt{3}a^{2};\;R_{c}=\sqrt{\frac{10\;+\;2\sqrt{5}}{4}}a$$

where $A_{f}$ is total surface area, a is edge length, and $R_{c}$ is the circumscribed radius (referred to as the diameter). The hexameric counts were then calculated using the total surface area and diameters of CsoS1A hexamers in a layer as reported previously (24).

10.1128/mbio.03629-21.9 TABLE S4 Absolute protein abundance per native and recombinant carboxysome based on 12-pentamer occupation and surface area coverage. *Based on surface area of ideal icosahedral with diameters measured from EM, 124.6 ± 9.6 nm (*n *= 272) and 131.8 ± 18.0 nm (*n *= 152) for native and recombinant carboxysomes, respectively. Values used for standardization are displayed in bold. Calculation is described in Materials and Methods in the main text. Download Table S4, DOCX file, 0.04 MB. Copyright © 2022 Sun et al. 2022 Sun et al. https://creativecommons.org/licenses/by/4.0/ This content is distributed under the terms of the Creative Commons Attribution 4.0 International license.

### Data availability.

The entire Skyline project and raw data for QconCAT quantification have been deposited at Panorama Public (72) with the access URL (https://panoramaweb.org/Wb6olk.url) and the ProteomeXchange identifier (ID) PXD031494. Raw LC-MS/MS data for label-free quantification have been deposited to the ProteomeXchange Consortium via the PRIDE (73) partner repository with the data set (https://www.ebi.ac.uk/pride/archive/projects/PXD031420). All other data are available from the corresponding author upon request.
