# Supplementary figures and images for: Decoding the Absolute Stoichiometric Composition and Structural Plasticity of α-Carboxysomes
Source: mBio. 2022 Mar 28;13(2):e03629-21. doi: 10.1128/mbio.03629-21 (PMC9040747; doi:10.1128/mbio.03629-21)

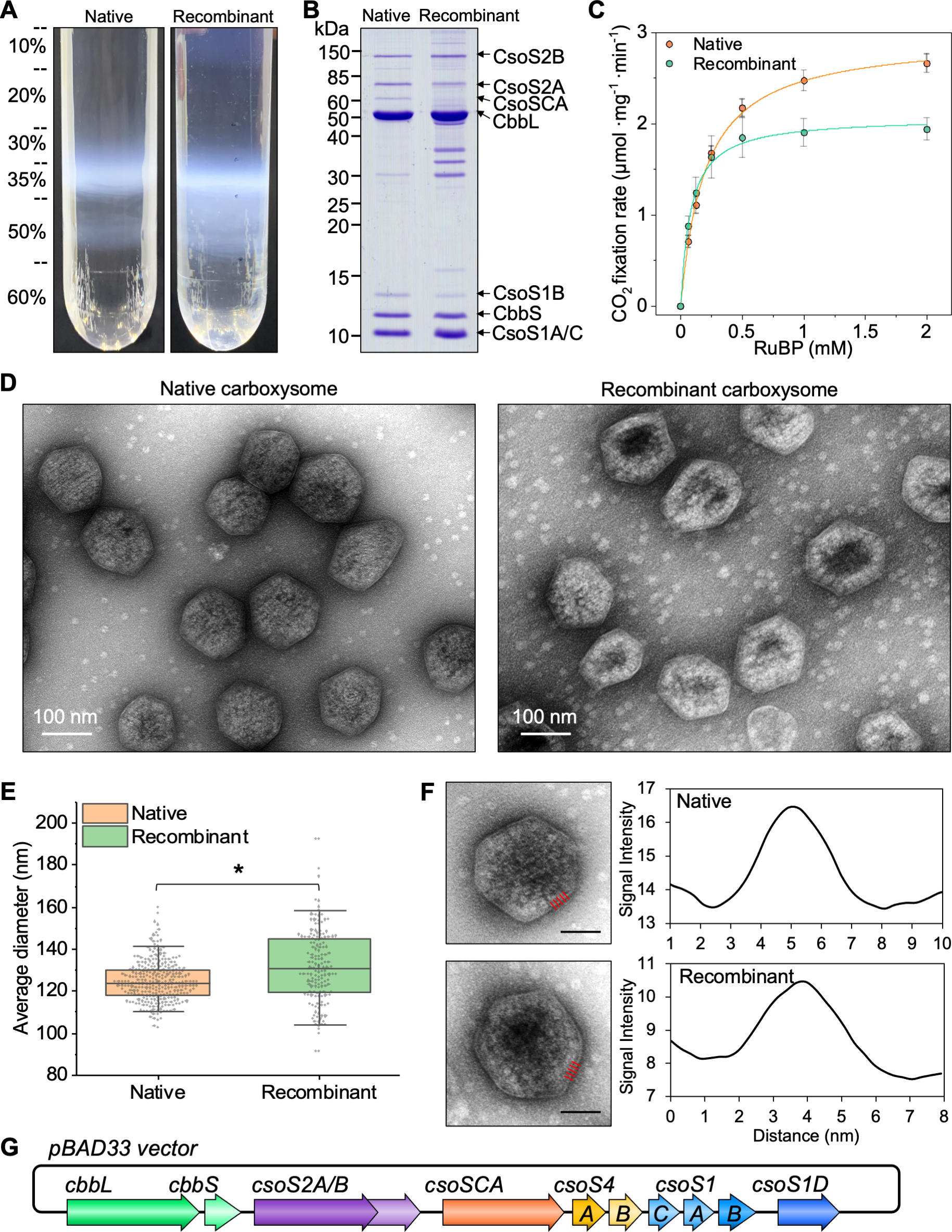

Supplement: FIG S1 [file mbio.03629-21-sf001.jpg]

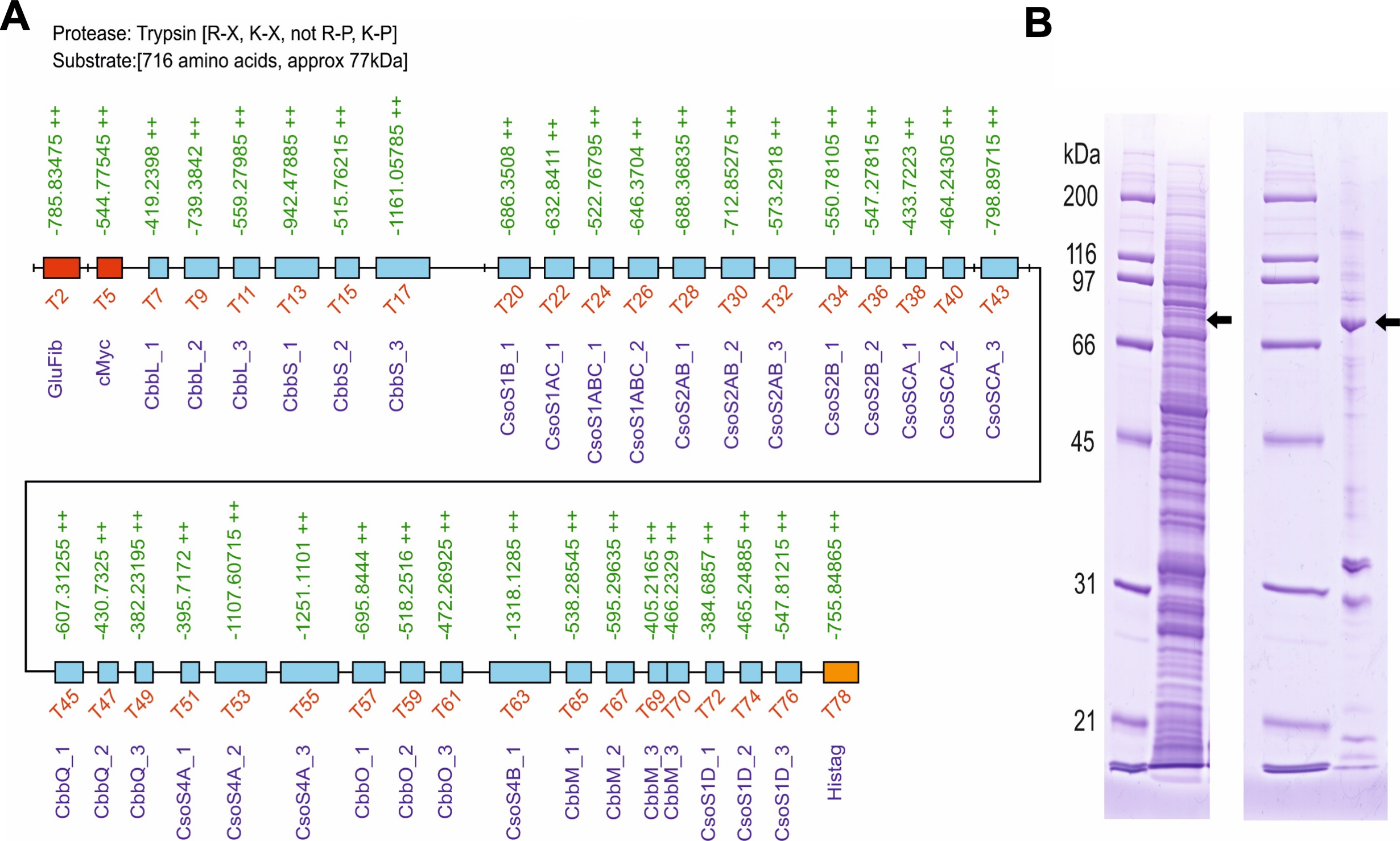

Supplement: FIG S2 [file mbio.03629-21-sf002.jpg]

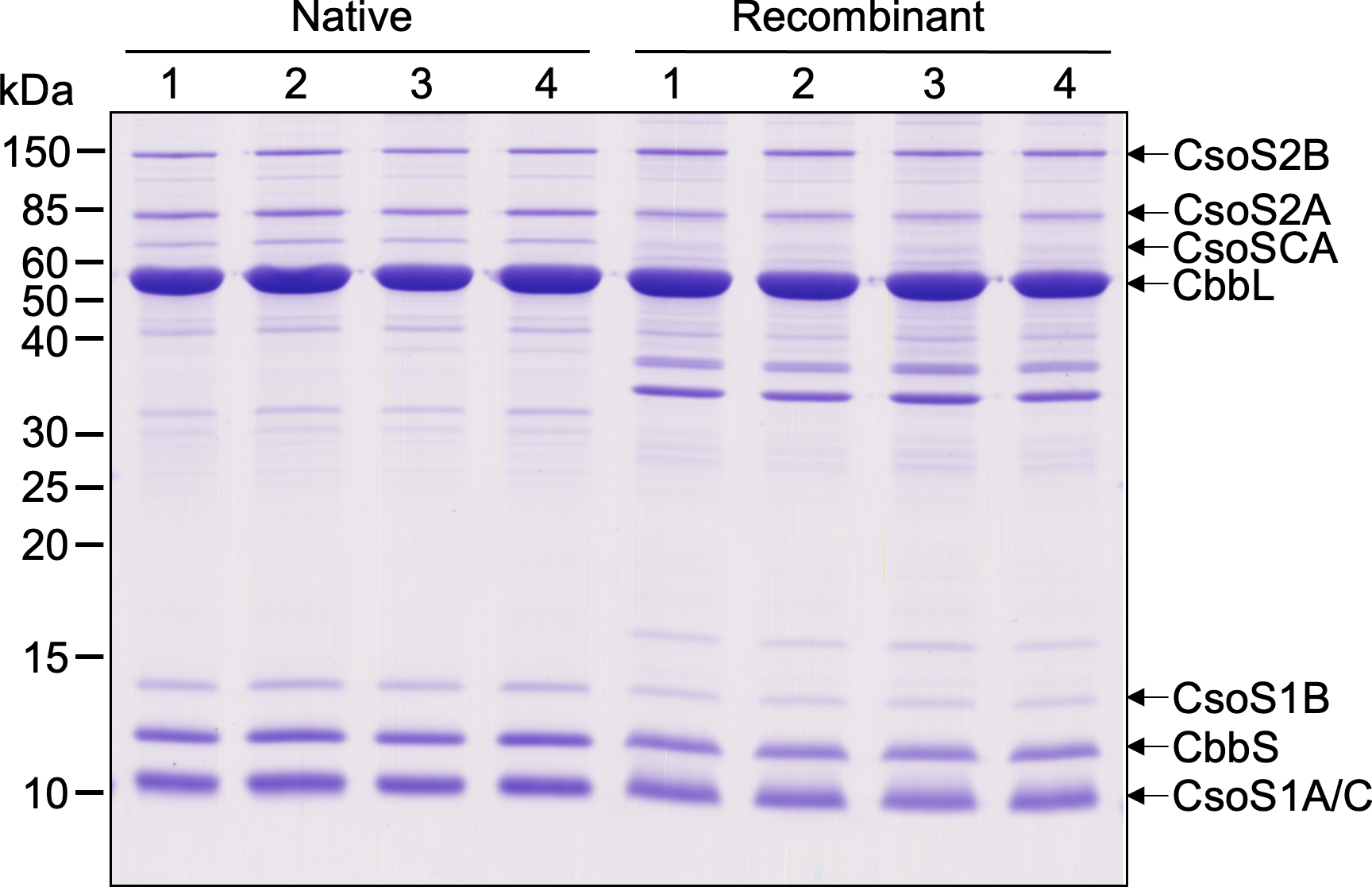

Supplement: FIG S3 [file mbio.03629-21-sf003.jpg]

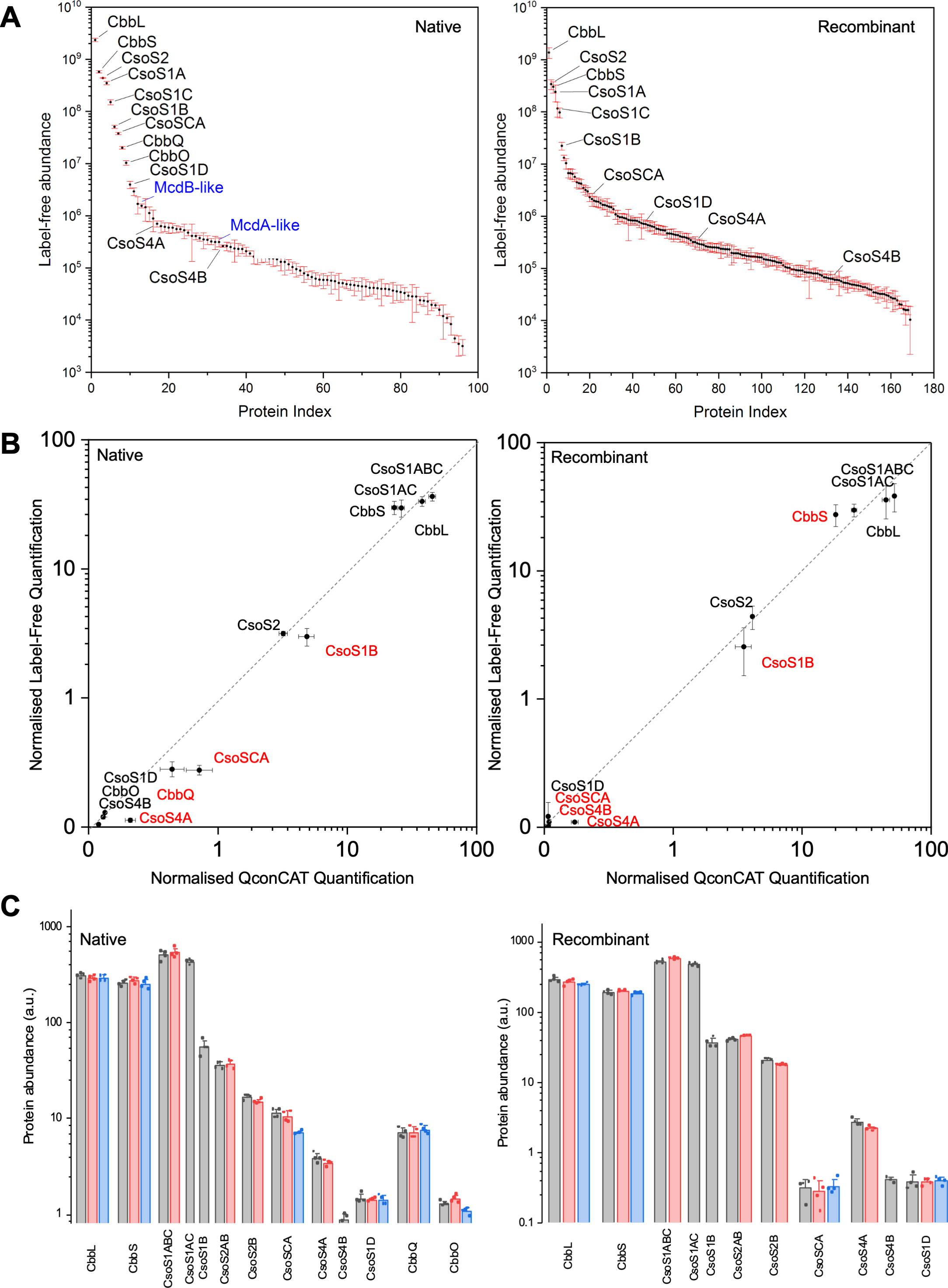

Supplement: FIG S4 [file mbio.03629-21-sf004.jpg]
