# Supplementary material for: Decoding the Absolute Stoichiometric Composition and Structural Plasticity of α-Carboxysomes
Source: mBio. 2022 Mar 28;13(2):e03629-21. doi: 10.1128/mbio.03629-21 (PMC9040747; doi:10.1128/mbio.03629-21)
Supplement: TABLE S1 [file mbio.03629-21-st001.docx]

**Table S1. Peptides derived from tryptic proteolysis of the QconCAT for carboxysome protein quantification.** The flanking sequences that recapitulate the true native primary sequence context, together with additional sequences that are derived from the loop assembly synthesis of the QconCAT are written in gray.

| **Peptide** | **M** | **[M+H]^+^** | **[M+2H]^++^** | **Sequence** | **Annotation** |
| --- | --- | --- | --- | --- | --- |
| T1 | 433 | 434 | 218 | MAGR | N-term |
| T2 | 1570 | 1571 | 786 | EGVNDNEEGFFSAR | GluFib |
| 3 | 356 | 357 | 179 | LPK |  |
| 4 | 403 | 404 | 203 | EQK |  |
| T5 | 1088 | 1089 | 545 | LISEEDLGGR | cMyc |
| 6 | 1164 | 1165 | 583 | GSQESSAEDVR | |
| T7 | 836 | 837 | 419 | FPLAYVK | CbbL_1 |
| 8 | 718 | 719 | 360 | TCGILR |  |
| T9 | 1477 | 1478 | 739 | LSGGDHLHTGTVVGK | CbbL_2 |
| 10 | 658 | 659 | 330 | LEGANR |  |
| T11 | 1117 | 1118 | 559 | VALEACVEAR | CbbL_3 |
| 12 | 686 | 687 | 344 | NQGQIK |  |
| T13 | 1883 | 1884 | 942 | YAIAQGWSPGIEHVEVK | CbbS_1 |
| 14 | 737 | 738 | 370 | NSMACR |  |
| T15 | 1030 | 1031 | 516 | SAYPTHQVK | CbbS_2 |
| 16 | 746 | 747 | 374 | LVAMWK |  |
| T17 | 2320 | 2321 | 1161 | LPFFGEQNVDNVLAEIEACR | CbbS_3 |
| 18 | 2398 | 2399 | 1200 | SAYSAAAAEMADVTGIALGMIETR |  |
| 19 | 599 | 600 | 301 | GLVVGR |  |
| T20 | 1371 | 1372 | 686 | SFVGGGYVTVMVR | CsoS1B_1 |
| 21 | 645 | 646 | 324 | GETIAR |  |
| T22 | 1264 | 1265 | 633 | VHSEVENILPK | CsoS1AC_1 |
| 23 | 682 | 683 | 342 | APQLVR |  |
| T24 | 1044 | 1045 | 523 | GETGAVNAAVR | CsoS1ABC_1 |
| 25 | 662 | 663 | 332 | AGACER |  |
| T26 | 1291 | 1292 | 646 | VGDGLVAAHIIAR | CsoS1ABC_2 |
| 27 | 655 | 656 | 329 | VHSGTR |  |
| T28 | 1375 | 1376 | 688 | AVPPKPQSQGGPGR | CsoS2AB_1 |
| 29 | 722 | 723 | 362 | NGYTLR |  |
| T30 | 1424 | 1425 | 713 | GTSVSGQQLDHAPK | CsoS2AB_2 |
| 31 | 636 | 637 | 319 | MSGTNK |  |
| T32 | 1145 | 1146 | 573 | GQSVTGNLVDR | CsoS2AB_3 |
| 33 | 1338 | 1339 | 670 | SELSAAYAEQNR | |
| T34 | 1100 | 1101 | 551 | ITGNDIAPSGR | CsoS2B_1 |
| 35 | 630 | 631 | 316 | ITGNAR |  |
| T36 | 1093 | 1094 | 547 | VVETSAFANR | CsoS2B_2 |
| 37 | 658 | 659 | 330 | NVPDSK |  |
| T38 | 865 | 866 | 434 | GFLNPYR | CsoSCA_1 |
| 39 | 750 | 751 | 376 | YVDNLK |  |
| T40 | 926 | 927 | 464 | GIFGYATAK | CsoSCA_2 |
| 41 | 431 | 432 | 217 | ALTK |  |
| 42 | 303 | 304 | 153 | ER |  |
| T43 | 1596 | 1597 | 799 | FSSLDEQNLLQFR | CsoSCA_3 |
| 44 | 631 | 632 | 317 | LSVGTR |  |
| T45 | 1213 | 1214 | 607 | WQDGPLTVAAR | CbbQ_1 |
| 46 | 661 | 662 | 332 | IGADMR |  |
| T47 | 859 | 860 | 431 | DALDTVVK | CbbQ_2 |
| 48 | 757 | 758 | 380 | TFFSTR |  |
| T49 | 762 | 763 | 382 | LLVYAGK | CbbQ_3 |
| 50 | 1229 | 1230 | 615 | LIASAAQAEVEK | |
| T51 | 789 | 790 | 396 | TLVSTNR | CsoS4A_1 |
| 52 | 688 | 689 | 345 | IADTNR |  |
| T53 | 2213 | 2214 | 1108 | IADMGHKPLLVVWEKPGAPR | CsoS4A_2 |
| 54 | 640 | 641 | 321 | QVAAPR |  |
| T55 | 2500 | 2501 | 1251 | QVAVDAIGCIPGDWVLCVGSSAAR | CsoS4A_3 |
| 56 | 631 | 632 | 317 | EAADAR |  |
| T57 | 1390 | 1391 | 696 | TGENPTLGALFDR | CbbO_1 |
| 58 | 686 | 687 | 344 | IALQSR |  |
| T59 | 1035 | 1036 | 518 | TDIPSSPYR | CbbO_2 |
| 60 | 720 | 721 | 361 | DDNMAR |  |
| T61 | 943 | 944 | 472 | ELGIALAEK | CbbO_3 |
| 62 | 1190 | 1191 | 596 | IQQSAASAETGK | |
| 63 | 2634 | 2635 | 1318 | VSVACDPIGVPEGCWVFTISGSAAR | |
| 64 | 703 | 704 | 353 | FGVPER |  |
| T65 | 1075 | 1076 | 538 | AIQLFDGPSK | CbbM_1 |
| 66 | 669 | 670 | 336 | DISHAK |  |
| T67 | 1189 | 1190 | 595 | IHDIYFPER | CbbM_2 |
| 68 | 644 | 645 | 323 | AIQSAR |  |
| T69 | 808 | 809 | 405 | YADLSLK | CbbM_3 |
| 70 | 930 | 931 | 466 | EEDLIAGGK |  |
| 71 | 751 | 752 | 377 | HILDVR |  |
| T72 | 767 | 768 | 385 | AFGNFGR | CsoS1D_1 |
| 73 | 732 | 733 | 367 | LTMNVR |  |
| T74 | 928 | 930 | 465 | LGEQVVER | CsoS1D_2 |
| 75 | 621 | 622 | 312 | AFGAEK |  |
| T76 | 1094 | 1095 | 548 | AAHVTLIDVR | CsoS1D_3 |
| 77 | 980 | 981 | 491 | AFGSAAGGSTR | |
| T78 | 1510 | 1511 | 756 | DQLALEHHHHHH* | HisTag |
