# Supplementary material for: Decoding the Absolute Stoichiometric Composition and Structural Plasticity of α-Carboxysomes
Source: mBio. 2022 Mar 28;13(2):e03629-21. doi: 10.1128/mbio.03629-21 (PMC9040747; doi:10.1128/mbio.03629-21)
Supplement: TABLE S2 [file mbio.03629-21-st002.docx]

**Table S2. DNA and protein sequences of the recombinant carboxysome QconCAT peptide.**

| **DNA sequences (2151 bp)** |
| --- |
| ATGGCAGGTCGTGAAGGTGTTAATGATAATGAAGAAGGCTTTTTTAGCGCACGTCTGCCGAAAGAACAGAAACTGATTAGCGAAGAAGATTTAGGCGGTCGCGGGTCTCAGGAGTCGAGTGCGGAGGATGTTCGTTTTCCGCTGGCATATGTTAAAACCTGTGGTATTCTGCGTCTTAGTGGTGGTGATCATCTGCATACCGGTACCGTTGTTGGTAAACTGGAAGGTGCCAACCGTGTGGCACTGGAAGCATGTGTTGAAGCACGTAATCAGGGTCAGATTAAATATGCGATCGCACAAGGCTGGAGCCCGGGTATTGAACATGTTGAAGTTAAAAATAGCATGGCATGTCGCAGTGCCTATCCGACCCATCAGGTTAAACTGGTTGCAATGTGGAAACTGCCGTTTTTCGGTGAACAGAATGTTGATAATGTTCTGGCAGAAATTGAAGCATGTCGTTCAGCCTATTCCGCTGCAGCAGCGGAGATGGCAGATGTTACCGGTATTGCACTGGGCATGATTGAAACCCGTGGTCTGGTTGTGGGTCGTAGCTTTGTTGGTGGTGGTTATGTTACCGTTATGGTTCGTGGTGAAACCATTGCCCGTGTGCATAGCGAAGTTGAAAATATTCTGCCGAAAGCACCGCAGTTAGTTCGCGGCGAGACAGGTGCAGTTAATGCAGCAGTTCGTGCAGGTGCATGTGAACGTGTTGGCGACGGCTTGGTTGCAGCACATATTATTGCACGCGTTCATTCAGGTACCCGTGCAGTTCCGCCAAAACCACAGAGCCAGGGTGGTCCGGGTCGTAATGGTTATACCCTGCGTGGTACCTCAGTTTCAGGTCAGCAGCTGGATCATGCACCGAAAATGAGCGGTACCAACAAGGGCCAAAGCGTTACCGGTAATCTGGTTGATCGTAGCGAACTGTCCGCTGCATACGCGGAGCAGAATCGTATTACCGGCAATGATATTGCACCGAGCGGTCGTATCACAGGTAATGCCAGAGTTGTTGAAACCAGCGCATTTGCAAATCGTAATGTTCCGGACAGCAAAGGCTTCCTGAATCCGTATCGTTATGTTGATAATCTGAAAGGTATTTTTGGCTATGCAACCGCAAAAGCACTGACCAAAGAACGTTTTAGCAGCCTGGATGAACAGAACCTTTTACAGTTTAGACTGAGCGTTGGTACCCGTTGGCAGGATGGTCCGCTGACAGTGGCCGCACGTATTGGTGCAGATATGCGTGATGCACTGGATACCGTTGTTAAAACTTTCTTCAGCACACGTTTACTGGTTTATGCAGGTAAACTGATTGCATCCGCTGCACAGGCGGAGGTTGAGAAAACCCTGGTTAGCACAAATCGTATTGCCGATACCAATCGCATTGCAGATATGGGTCATAAACCGCTGCTGGTTGTTTGGGAAAAACCGGGTGCACCGCGTCAGGTTGCCGCCCCTCGCCAGGTGGCAGTTGATGCAATTGGTTGTATTCCGGGTGATTGGGTTCTGTGCGTTGGTAGCAGCGCAGCACGTGAAGCAGCAGATGCACGTACCGGTGAAAATCCGACCCTGGGTGCCCTGTTTGATCGTATTGCACTGCAGAGCCGTACAGATATTCCTAGTAGTCCTTATCGTGATGATAATATGGCACGTGAACTGGGTATTGCCCTTGCAGAGAAAATTCAGCAGTCCGCTGCATCGGCGGAGACAGGTAAAGTTAGCGTTGCATGTGATCCGATTGGCGTTCCTGAAGGTTGCTGGGTGTTCACCATTAGCGGTAGCGCAGCACGTTTTGGTGTTCCGGAACGTGCCATCCAGTTGTTCGATGGTCCGAGCAAAGATATTAGCCATGCAAAAATTCATGATATTTATTTCCCTGAGCGTGCAATTCAGAGCGCCCGTTATGCAGATCTGAGTCTGAAAGAGGAAGATCTGATTGCAGGTGGTAAACATATTCTGGATGTTAGAGCATTTGGTAACTTTGGTCGCCTGACCATGAATGTTCGTCTGGGTGAACAGGTTGTTGAACGTGCATTTGGTGCAGAAAAAGCAGCACATGTTACCCTGATTGATGTTCGCGCCTTTGGTTCCGCTGCAGGTGGATCTACTAGAGACCAGCTGGCACTGGAACATCATCATCACCATCACTAA |
| **Protein sequence (716 AA)** |
| MAGREGVNDNEEGFFSARLPKEQKLISEEDLGGRGSQESSAEDVRFPLAYVKTCGILRLSGGDHLHTGTVVGKLEGANRVALEACVEARNQGQIKYAIAQGWSPGIEHVEVKNSMACRSAYPTHQVKLVAMWKLPFFGEQNVDNVLAEIEACRSAYSAAAAEMADVTGIALGMIETRGLVVGRSFVGGGYVTVMVRGETIARVHSEVENILPKAPQLVRGETGAVNAAVRAGACERVGDGLVAAHIIARVHSGTRAVPPKPQSQGGPGRNGYTLRGTSVSGQQLDHAPKMSGTNKGQSVTGNLVDRSELSAAYAEQNRITGNDIAPSGRITGNARVVETSAFANRNVPDSKGFLNPYRYVDNLKGIFGYATAKALTKERFSSLDEQNLLQFRLSVGTRWQDGPLTVAARIGADMRDALDTVVKTFFSTRLLVYAGKLIASAAQAEVEKTLVSTNRIADTNRIADMGHKPLLVVWEKPGAPRQVAAPRQVAVDAIGCIPGDWVLCVGSSAAREAADARTGENPTLGALFDRIALQSRTDIPSSPYRDDNMARELGIALAEKIQQSAASAETGKVSVACDPIGVPEGCWVFTISGSAARFGVPERAIQLFDGPSKDISHAKIHDIYFPERAIQSARYADLSLKEEDLIAGGKHILDVRAFGNFGRLTMNVRLGEQVVERAFGAEKAAHVTLIDVRAFGSAAGGSTRDQLALEHHHHHH |
