# Supplementary material for: Decoding the Absolute Stoichiometric Composition and Structural Plasticity of α-Carboxysomes
Source: mBio. 2022 Mar 28;13(2):e03629-21. doi: 10.1128/mbio.03629-21 (PMC9040747; doi:10.1128/mbio.03629-21)
Supplement: TABLE S3 [file mbio.03629-21-st003.docx]

**Table S3. Calculation of carboxysome surface area, shell hexamer content, and carboxysome diameter.** *CsoS1 and CsoS4 width/area obtained from previous publications ([3](#_ENREF_3), [4](#_ENREF_4)). ^&^Assumed packing densities of 74% (Kepler packing) ([5](#_ENREF_5)) in the proposed carboxysome model based on the measured carboxysome diameters.

| **Parameter** | **native α-carboxysome**  **(*n* = 272)** | **recombinant α-carboxysome**  **(*n* = 152)** |
| --- | --- | --- |
| EM-measured diameter (nm) | 124.6 ± 9.6 | 131.8 ± 18.0 |
| Radius of circumscribed sphere (nm) | 62.3 ± 4.8 | 65.9 ± 9.0 |
| Facet side length (nm) | 65.5 ± 5.0 | 69.3 ± 9.4 |
| Carboxysome surface area (nm^2^) | 37407.3 ± 5864.6 | 42354.8 ± 11745.1 |
| CsoS1 hexamer width (nm)* | 6.6 | 6.6 |
| CsoS1 hexamer area (nm^2^)* | 38.9 | 38.9 |
| CsoS4 pentamer area (nm^2^)* | 30.3 | 30.3 |
| All facet hexamer counts | 977.6 ± 150.9 | 1080.2 ± 302.1 |
| Estimated Rubisco counts per carboxysome^&^ | 410.7 ± 101.8 | 491.4 ± 216.1 |
