# Supplementary material for: Decoding the Absolute Stoichiometric Composition and Structural Plasticity of α-Carboxysomes
Source: mBio. 2022 Mar 28;13(2):e03629-21. doi: 10.1128/mbio.03629-21 (PMC9040747; doi:10.1128/mbio.03629-21)
Supplement: TABLE S5 [file mbio.03629-21-st005.docx]

**Table S5. Primer sets used for pBAD33-CBS1D construction.**

| **Primer** | **Sequence (5’-3’)** |
| --- | --- |
| S1D-R | GCTACGCCTGAATAAGTGCTGCAGGCGGCCCTGTTCGACTTAAGCATTATGGCGGCCGCTTAGAACCCTTCAGCGCGACGCG |
| S1D-F | GTTTAACTTTAAGAAGGAGATATACAATGGCAGTTAAAAAGTATAGTGCTGGTG |
| pBAD33-R | TGTATATCTCCTTCTTAAAGTTAAACAAAATTATTTCTAGAGG |
| pBAD33-F | GCACTTATTCAGGCGTAGCAAC |
