# Supplementary material for: Decoding the Absolute Stoichiometric Composition and Structural Plasticity of α-Carboxysomes
Source: mBio. 2022 Mar 28;13(2):e03629-21. doi: 10.1128/mbio.03629-21 (PMC9040747; doi:10.1128/mbio.03629-21)
Supplement: TABLE S4 [file mbio.03629-21-st004.docx]

**Table S4. Absolute protein abundance per native and recombinant carboxysome based on 12-pentamer occupation and surface area coverage.** *Based on surface area of ideal icosahedral with diameters measured from EM, 124.6 ± 9.6 nm (*n* = 272) and 131.8 ± 18.0 nm (*n* = 152) for native and recombinant carboxysomes, respectively. Values used for standardization were displayed in bold. Calculation was described in Methods.

| **Protein** | **Native α-carboxysome** | | **Recombinant α-carboxysome** | |
| --- | --- | --- | --- | --- |
|  | **12 pentamers** | **CsoS1 coverage*** | **12 pentamers** | **CsoS1 coverage*** |
| CbbL | 490 ± 21 | 447 ± 19 | 717 ± 53 | 426 ± 32 |
| CbbS | 433 ± 21 | 395 ± 19 | 515 ± 20 | 306 ± 12 |
| CsoS1AC | 946 ± 69 | **863 ± 63** | 1684 ± 75 | **1001 ± 45** |
| CsoS1B | 123± 27 | **112 ± 25** | 133 ± 16 | **79 ± 9** |
| CsoS2A | 272 ± 52 | 248 ± 47 | 513 ± 16 | 305 ± 9 |
| CsoS2B | 210 ± 16 | 192 ± 15 | 419 ± 21 | 249 ± 13 |
| CsoSCA | 64 ± 4 | 58 ± 4 | 3 ± 1 | 2 ± 1 |
| CsoS4A | **10 ± 0** | 9 ± 0 | **11 ± 1** | 6 ± 1 |
| CsoS4B | **2 ± 0** | 2 ± 0 | **1 ± 1** | 1 ± 1 |
| CsoS1D | 3 ± 0 | **3 ± 0** | 1 ± 0 | **1 ± 0** |
| CbbQ | 16 ± 1 | 15 ± 1 | N/A | N/A |
| CbbO | 17 ± 1 | 15 ± 1 | N/A | N/A |
